# Natural Products Biosynthesis by *Streptomyces netropsis* IMV Ac-5025 under Exogenous Sterol Action

**DOI:** 10.3390/antibiotics13020146

**Published:** 2024-02-01

**Authors:** Mariia Loboda, Liudmyla Biliavska, Galyna Iutynska, Jake Newitt, Ruslan Mariychuk

**Affiliations:** 1 Department of General and Soil Microbiology, D.K. Zabolotny Institute of Microbiology and Virology, National Academy of Sciences of Ukraine, Akademika Zabolotnoho Str., 154, 03143 Kyiv, Ukraine; marichka20loboda@gmail.com (M.L.); bilyuvskal@ukr.net (L.B.); galyna.iutynska@gmail.com (G.I.); 2 Department of Molecular Microbiology, John Innes Centre, Norwich NR4 7UH, UK; jake.newitt@jic.ac.uk; 3 Department of Ecology, Faculty of Humanities and Natural Science, University of Presov, 08001 Presov, Slovakia

**Keywords:** *Streptomyces netropsis*, polyene antibiotics, β-sitosterol, sterols, phytohormones, auxins, cytokinins

## Abstract

Streptomycetes are known as producers of bioactive substances, particularly antibiotics. *Streptomyces netropsis* IMV Ac-5025 simultaneously produces different classes of antibiotics, including polyene compounds, phytohormones, and sterols, but the metabolic pathways involved in their biosynthesis are largely understudied. The aim of this work was to explore the biosynthesis of polyene antibiotics, sterols, and phytohormones when the producer is cultivated in a nutrient medium supplemented with exogenous β-sitosterol. Gas chromatography and high-performance liquid chromatography were applied to analyze the spectrum of bioactive compounds. The obtained results demonstrated not only an increase in the accumulation of biomass but also polyene antibiotics, intracellular sterols, auxins, and cytokinins, when cultivating *S. netropsis* IMV Ac-5025 in a liquid medium with the addition of β-sitosterol. The amount of biomass raised 1.5–2-fold, whilst the sum of polyene antibiotics increased 4.5-fold, sterols’ sum (ergosterol, cholesterol, stigmasterol, β-sitosterol, and 24-epibrassinolide) by 2.9-fold, auxins’ sum (indole-3-acetic acid, indole-3-acetic acid hydrazide, indole-3-carbinol, indole-3-butyric acid, indole-3-carboxaldehyde, and indole-3-carboxylic acid) by 6-fold, and cytokinins’ sum (zeatin, isopentyladenine, zeatin riboside, and isopentenyladenosine) by 11-fold. Thus, we put forward the hypothesis that β-sitosterol plays a regulatory role in the network of biosynthetic reactions of *S. netropsis* IMV Ac-5025.

## 1. Introduction

*Actinobacteria*, including *Streptomyces* species, are important for research due to their ability to produce biologically active secondary metabolites with various properties and their significant role in the soil microbiome [1]. Having large chromosomes (several million bp), they are characterized by the ability to simultaneously produce a wide range of biological substances: antibiotics, sterols, fatty acids, phytohormones, vitamins, etc. These secondary metabolites are important for interacting with other microorganisms in microbial biocenosis. Building upon this, it is noteworthy to mention another significant process related to the biosynthesis of these bioactive metabolites. It involves the symbiotic relationship between representatives of the *Streptomyces* genus and plants through direct antimicrobial activity, the induction of plant immunity by indirect biosynthetic pathways, and the improvement of plant growth through the secretion of phytohormones. They protect plants against phytopathogenic bacteria and fungi, as well as phytoparasitic nematodes and other stress factors [2]. These metabolites may act as signaling molecules by affecting the expression of genes unrelated to stress [1,2]. Streptomycetes are well-studied for their ability to synthesize antibiotics of various classes, which exhibit antibacterial, antifungal, antiviral, antitumor, antihypertensive, and immunosuppressive effects. These include polyene antibiotics (nystatin and amphotericin B, mediomycins A, B, clethramycin, candidine), aminoglycosides (streptomycin and neomycin), macrolides (erythromycin), tetracyclines (tetracycline and oxytetracycline), glycopeptides (vancomycin), and beta-lactams (although less common, some species produce beta-lactam antibiotics, such as cephalosporins) [3]. These antibiotics have diverse mechanisms of action: the induction of membrane pore formation and ion leakage, the inhibition of protein biosynthesis or DNA replication, the destruction of biological membranes or cell walls, and changes in the metabolism of sensitive cells [4]. Polyene antibiotics synthesized by streptomycetes have caught the attention of researchers for several reasons. First, their structure and biosynthesis have not been sufficiently studied. Second, target microorganisms are rarely resistant to these antibiotics [5]. Therefore, the study of the biosynthesis of polyene antibiotics by streptomycetes is important for industrial biotechnology.

Sterols are important components of cell membranes and are well studied in eukaryotes, particularly their influence on the permeability and plasticity of membranes and the formation of lipid rafts. In bacteria, sterols perform a similar function, except for the formation of functional membrane microdomains [6]. Although some soil streptomycetes are able to accumulate sterols, their role in secondary metabolism remains unclear, especially in the biosynthesis of polyene antibiotics and other bioactive metabolites. As a result of the most recent data, we can only assume a potential regulatory role and view their biosynthesis as an evolutionary process. The presence of sterol biosynthesis genes in bacteria suggests that these pathways are ancient and that bacteria played a fundamental role in the origin of sterol biosynthetic pathways. This could potentially have implications for the evolution of antibiotic resistance [4]. Bacteria acquired this biosynthesis pathway from ancient eukaryotes, perhaps through horizontal gene transfer due to the sporadic and sparse distribution of sterol synthesis in bacteria [7,8].

Studies of the functional role of sterols in bacteria are limited; the results are mixed, and they range from essential for viability (*Gemmata obscuriglobus)* to inconsequential for normal physiology and metabolome (*Sigmatella aurantica*). Phylogenetic studies suggest that bacteria play a fundamental role in sterol biosynthesis for other bacterial species [7]. Sterols are tetracyclic triterpenoid lipids derived from steroids that contain a hydroxyl group in the third position of the molecule. They are integral components of plasma membranes and involved in adaptive reactions, play a signaling role, and impact membrane permeability. Aerial hyphae and spores of *Streptomyces* bacteria contain sterols that reduce water loss across the membrane [9]. The ability of some bacteria to synthesize sterols is associated with survival in extreme conditions such as high temperatures, pH, salinity, etc. [10]. The lipid bilayer of the plasma membrane is thought to be compartmentalized by the presence of specific microdomains. They are composed of sterols and sphingolipids, commonly known as “lipid rafts”, and are believed to exist in eukaryotic cells. However, there have also been reports on the presence of sterol-mediated microdomains in bacterial cell membranes [11]. The microdomains in the plasma membrane of streptomycetes remain poorly understood despite increasing attention being given to them. In contrast to the bulk of the membrane, microdomains contain more saturated fatty acids, sterols, sphingolipids, and phospholipids. They may include sphingosterols, cholesterol, sitosterol, stigmasterol, and 24-methylcholesterol. Sterols and their derivatives can change the properties and number of “lipid rafts”, as well as their function [11]. In general, their discovery in bacteria reveals a new level of complexity in signal transmission and membrane organization. The ability of *S. netropsis* IMV Ac-5025 to synthesize sterols may potentially indicate the existence of “lipid rafts” in the producer’s cells.

β-Sitosterol is characterized by high bioactivity among sterols. It is a plant-derived phytosterol, also produced by other organisms. Some of its key activities include anti-inflammatory properties, antioxidant activity, cholesterol-lowering effects, immune system modulation, and anti-cancer potential [12]. β-sitosterol demonstrates antibacterial activity against *Staphylococcus aureus* and *Escherichia coli* [13], yet the role of β-sitosterol in the metabolism of bacterial cells remains poorly understood. It is known that polyene antibiotics interact with sterols in the cell membranes of pathogenic microorganisms and change the permeability of the membranes of a number of bacteria, which leads to the leakage of important cellular components and, ultimately, to the lysis and death of the cell [14]. Some authors determined that filipin, amphotericin B, nystatin, etruscomycin, and pimaricin affect the permeability of *Acholeplasma laidlawii* cells [15]. Filipin disrupted the structure of the membrane after interacting with cholesterol and caused the release of both small ions, such as K^+^, and large protein molecules, such as glucose-6-phosphate dehydrogenase. Amphotericin B, nystatin, and etruscomycin created water pores of a certain size in the membrane after interacting with cholesterol. Pimaricin did not change the permeability of *A. laidlawii* [15,16,17]. As was demonstrated, polyene antibiotics can affect membrane sterols in a wide variety of ways, from slowing their rotation axially to completely immobilizing them in the membrane and forming “hard” complexes. In bacteria capable of producing polyene antibiotics, the interaction of polyene antibiotics with sterols remains unexplored. Also, the effect of sterols on the synthesis of biologically active substances—antibiotics, phytohormones, and sterols—has not been studied.

The *Streptomyces netropsis* IMV Ac-5025 strain was isolated by researchers of the D.K. Zabolotny Institute of Microbiology and Virology of the National Academy of Sciences of Ukraine, Kyiv, Ukraine. It simultaneously produces a complex of bioactive substances, including polyene antibiotics (candidine and tetraene compound), auxins (indole-3-acetic acid, indole-3-acetic acid hydrazide, indole-3-carbinol, indole-3-butyric acid, indole-3-carboxaldehyde, and indole-3-carboxylic acid), cytokinins (zeatin, isopentyladenine, zeatin riboside, and isopentenyladenosine), gibberellins, abscisic acid, amino acids, phospholipids, monoglycerides and diglycerides, fatty acids (isopalmitic, heptadecene, stearic, oleic, and arachidonic, etc.), and sterols (squalene, ergosterol, cholesterol, stigmasterol, β-sitosterol, and 24-epibrassinolide) [9,18]. The bioctivity of polyene compounds has been measured against representatives of *Alternaria*, *Fusarium*, and *Cladosporium* genera [19]. The obtained experimental results showed that the complex of these metabolites promotes the growth of agricultural crops in different climatic zones, increases their yield, and improves product quality. It occurs by activating the expression of genes that control plants’ growth and development. They also accelerate the de novo biosynthesis of cytoplasmic mRNAs encoding the biosynthesis of structural and regulatory proteins that restore stress-induced metabolic disturbances in plant cells [20]. Other investigations showed that the metabolite complex of *S. netropsis* IMV Ac-5025 significantly increased the resistance of plants to biotic stresses (phytopathogenic fungi and plant-parasitic nematodes) by inducing RNAi processes in plant cells [20,21]. Therefore, we performed factor analysis to determine the relationship between the biosynthesis of polyene antibiotics and other classes of metabolites. Polyene antibiotic biosynthesis had the highest correlation with the biosynthesis of phytohormones and sterols out of all the bioactive substances (BAS) synthesized by the strain: isopentenyladenosine (cytokinin) with a factor loading strength of 80%, indole-3-carbinol (auxin) at 80%, and β-sitosterol at 70% [18]. Studying the factors affecting this strain’s biosynthetic capacity is therefore important from both a metabolic perspective in the producer cell and from a practical perspective of its application.

The present study aimed to analyze the effect of exogenous β-sitosterol on the biosynthetic capacity of the *S. netropsis* IMV Ac-5025 strain by measuring the accumulation of polyene antibiotics and other secondary metabolites, such as sterols, auxins, and cytokinins. The influence of exogenous β-sitosterol on the accumulation of biomass was also explored.

## 2. Results

### 2.1. Biosynthesis of Polyene Antibiotics

**Figure 1:**
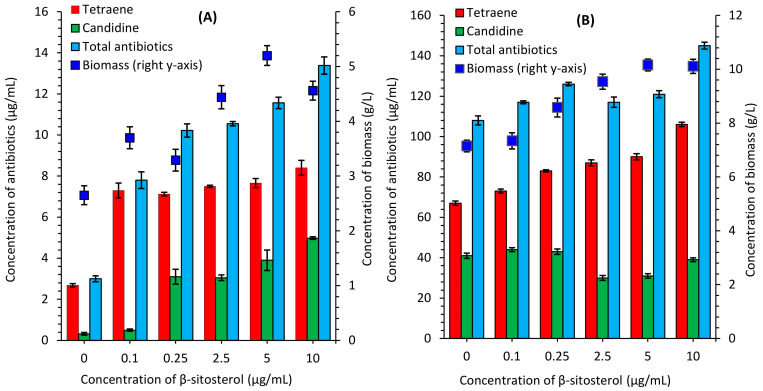
Polyene antibiotics accumulation by *S. netropsis* IMV Ac-5025 grown in synthetic (**A**) and organic (**B**) nutrient media in culture liquid supplemented with exogenous β-sitosterol. The secondary axis shows the amount of biomass of streptomycete (blue squares) after the cultivation in a liquid nutrient medium (g/L). In terms of intracellular metabolites, this makes it possible to analyze the biosynthetic activity of the strain as the amount of antibiotic in the culture liquid is a changeable parameter determined by the amount of biomass. Knowing the value of the last one, we can always calculate the amount of antibiotic in 1 g or 1 mg of cells, etc.

Polyene antibiotics were quantified in *S. netropsis* IMV Ac-5025, which was grown both in synthetic and organic nutrient media supplemented with exogenous β-sitosterol. The addition of β-sitosterol increased both the accumulation of heptaene candidine and tetraene antibiotics and their excretion from the cells into the culture liquid [22]. When the strain was cultivated in a synthetic nutrient medium, the amount of tetraene increased from 2.68 μg/mL to 8.4 μg/mL, and the amount of candidine increased from 0.32 μg/mL to 4.98 μg/mL, equivalent to a 3.1-fold and 16-fold increase relative to the control. The same effect was observed when the strain was cultivated in an organic nutrient medium. The amount of tetraene increased from 67 μg/mL to 106 μg/mL, equivalent to a 1.6-fold increase. However, the amount of candidine fluctuated inconsistently. The sum of polyene antibiotics ranged between 3 μg/mL and 13.38 μg/mL in the synthetic nutrient medium and between 108 μg/mL and 145 μg/mL in the organic nutrient medium. The greatest increase in production was observed with the addition of 10 μg/mL of β-sitosterol. This condition yielded 4.5- and 1.3-fold higher production for the synthetic and organic nutrient media, respectively, when compared to the controls (Figure 1, Table S1). The biomass increased from 2.65 g/L to 4.56 g/L and from 7.15 g/L to 10.11 g/L, depending on the type of nutrient medium. Media supplementation with 5 μg/mL of β-sitosterol led to a 1.7-fold increase in biomass accumulation in the synthetic nutrient medium and a 1.5-fold increase in the nutrient organic medium.

**Figure 2:**
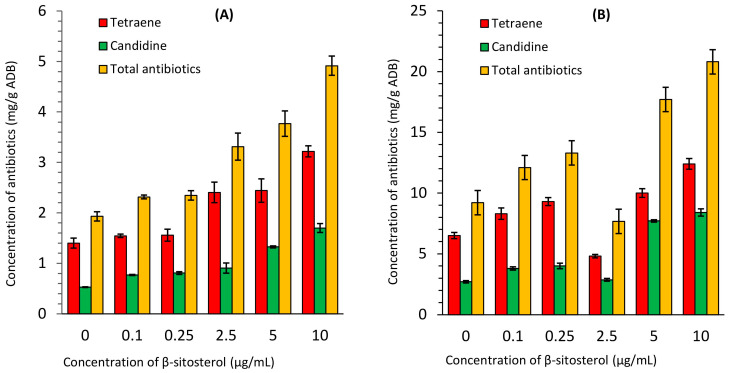
Accumulation of polyene antibiotics in *S. netropsis* IMV Ac-5025 biomass grown in synthetic (**A**) and organic (**B**) nutrient media supplemented with β-sitosterol.

The accumulation of polyene antibiotics in the biomass of *S. netropsis* IMV Ac-5025 increased when supplemented with β-sitosterol (Figure 2, Table S1). Their sum ranged between 1.929 mg/g and 4.917 mg/g ADB in the synthetic nutrient medium and from 9.210 mg/g to 20.8 mg/g ADB in the organic nutrient medium. In the synthetic nutrient medium, the content of tetraene ranged between 1.399 mg/g and 3.218 mg/g ADB and increased 2.3-fold; candidine increased from 0.53 mg/g to 1.699 mg/g ADB and increased 3.2-fold. Their sum rose by 2.5-fold when 10 μg/mL of β-sitosterol was added. In the organic nutrient medium, the amount of tetraene varied between 6.5 mg/g and 12.4 mg/g ADB and increased 1.9-fold; candidine changed from 2.71 mg/g to 8.4 mg/g ADB and rose by 3.1-fold. Their sum rose by 2.3-fold when 10 μg/mL of β-sitosterol was added. An increase in the accumulation of polyene candidine and tetraene antibiotics in producer cells was calculated per 1 g of absolutely dry biomass. This is convincing evidence that exogenous β-sitosterol not only affects cell division, but also the biosynthetic activity of the producer.

### 2.2. Biosynthesis of Sterols

**Figure 3:**
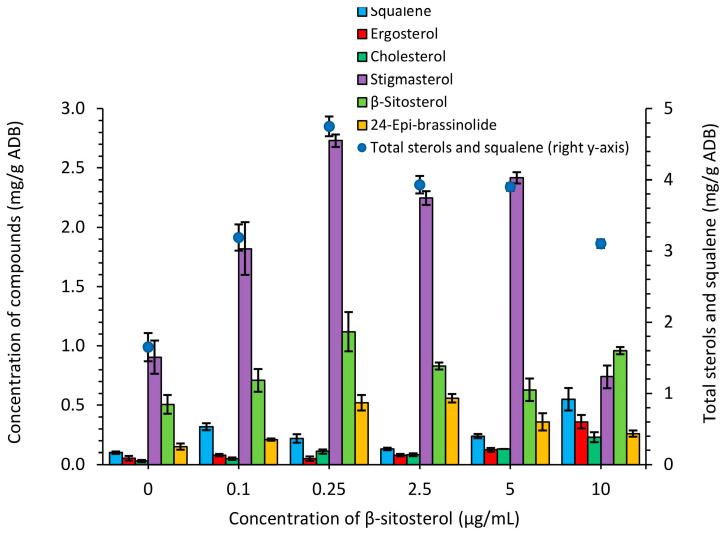
Accumulation of squalene and sterols in *S. netropsis* IMV Ac-5025 biomass grown in synthetic nutrient medium supplemented with exogenous β-sitosterol. Blue circles show the sum of sterols and squalene. For clarity, this parameter is displayed on the secondary axis as these values significantly exceed the number of separated sterols.

The biosynthesis of sterols and squalene in the *S. netropsis* IMV Ac-5025 biomass was measured after it was grown in a synthetic nutrient medium supplemented with exogenous β-sitosterol. The producer was found to be capable of synthesizing intracellular squalene and sterols: ergosterol, cholesterol, stigmasterol, β-sitosterol, and 24-epibrassinolid [22]. At the same time, there was an increase in their accumulation under the action of β-sitosterol (Figure 3, Table S2).

The sum of sterols, including squalene, ranged between 1.65 mg/g and 4.750 mg/g ADB and was highest when 0.25 µg/mL of β-sitosterol was added, with a 2.9-fold increase relative to the control. Squalene is a polyunsaturated hydrocarbon an acyclic triterpene with six double bonds. It is an important intermediate in biosynthesis sterols and hopanoids. Sterol metabolism begins with its biosynthesis and further conversions [23]. Its content ranged between 0.102 mg/g and 0.50 mg/g ADB and was the highest when 10 μg/mL of β-sitosterol was applied. Ergosterol regulates the membrane fluidity and shape while also acting as an antifungal target. The quantity ranged between 0.1 mg/g and 0.55 mg/g ADB, with the highest concentration present in cultures supplemented with 10 μg/mL of exogenous β-sitosterol [24]. Cholesterol is a type of lipid and also serves as a structural component of cell membranes and a precursor for the biosynthesis of steroid hormones and vitamin D. Numerous microorganisms are known to produce cholesterol, including streptomycetes [3]. The intracellular accumulation of cholesterol by *S. netropsis* IMV Ac-5025 ranged between 0.03 mg/g and 0.23 mg/g ADB and increased 6.9-fold compared to the control when supplemented with 10 µg/mL of β-sitosterol (Figure 3, Table S2).

Stigmasterol is a structural component of the lipid core of cell membranes and a precursor of numerous secondary metabolites—including brassinosteroids, plant steroid hormones that play an important role in plant growth and development—as well as mediating plant responses to various stresses. Stigmasterol is synthesized from β-sitosterol by a single reaction [25,26]. The amount of stigmasterol ranged between 0.74 mg/g and 2.736 mg/g ADB, and with the addition of β-sitosterol, it ranged between 0.51 mg/g and 1.12 mg/g ADB. Supplementation with 0.25 μg/mL of β-sitosterol led to a 3-fold increase in stigmasterol and a 2.2-fold increase in β-sitosterol (Figure 3, Table S2).

24-Epibrassinolide is a type of brassinosteroid; it is involved in the regulation of cell elongation and division and has been shown to improve plant fitness in salt- and nickel-stressed soils, as well as to enhance an antioxidant enzyme activity (SOD, APX, CAT). It contributes to plant resistance through improvements in photosynthesis and the protection of chloroplast ultrastructure, among others [27]. While studies have demonstrated a role in resisting drought stress in plants, little is known about how it functions in streptomycetes. Moreover, it has been demonstrated that a few Actinobacteria species are capable of synthesizing this phytohormone. The quantity of 24-epibrassinolide ranged between 0.15 mg/g and 0.56 mg/g ADB and increased 3.8-fold in the *S. netropsis* IMV Ac-5025 biomass when supplemented with 0.25 μg/mL of β-sitosterol (Figure 3, Table S2). The ability of the producer to accumulate 24-epibrassinolide has great value for application in plant protection strategies.

### 2.3. Biosynthesis of Auxins

**Figure 4:**
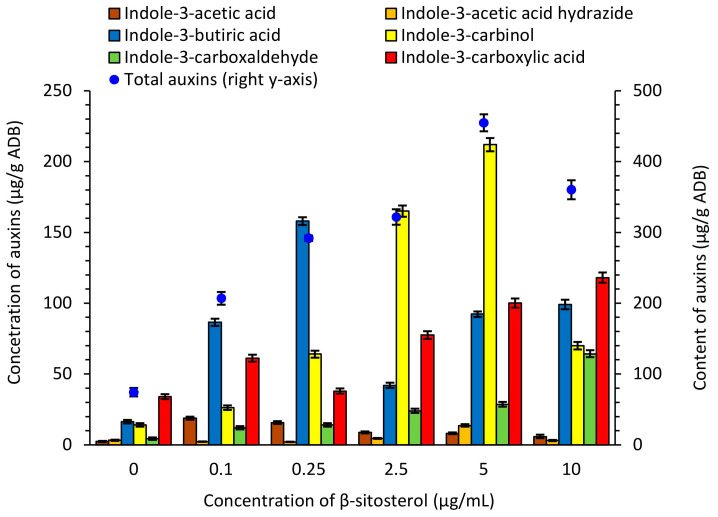
Biosynthesis of auxins in *S. netropsis* IMV Ac-5025 biomass grown in synthetic nutrient medium supplemented with exogenous β-sitosterol. Blue circles show the sum of auxins. For greater clarity, this parameter is displayed on the secondary axis as these values significantlyexceed the number of separated auxins.

In light of the correlation analysis we conducted previously, it was expedient to examinethe the biosynthesis of auxins by *S. netropsis* IMV Ac-5025 supplemented with exogenous β-sitosterol [18]. All aspects of cell growth and development depend on maintaining adequate levels of active auxins. Their transport, biosynthesis, and interconversion of modified forms can affect the cellular auxins levels [28,29]. As secondary metabolites, they are synthesized in the stationary phase of growth by most soil bacteria, both associated with and not associated with plants. The biosynthesis of auxins was measured in the *S. netropsis* IMV Ac-5025 biomass, which was grown in a synthetic nutrient medium supplemented with exogenous β-sitosterol. The strain was found to be capable of synthesizing indole-3-acetic acid (IAA), indole-3-acetic acid hydrazide, indole-3-carbinol, indole-3-butyric acid, indole-3-carboxaldehyde, and indole-3-carboxylic acid (Figure 4, Table S3). The sum of auxins ranged between 74.244 μg/g and 454.801 μg/g ADB and was highest when 5 µg/mL of β-sitosterol was added, with a 6-fold increase relative to the control.

Indole-3-carbinol and indole-3-butyric acid accumulated to greater extents in the producer’s biomass. Indole-3-carbinol is a signaling molecule modulating different cellular and developmental pathways. This phytohormone has also been found to inhibit the growth of various cancer cells and, additionally, to inhibit the growth of some pathogens [30]. Thus, it has remarkable potential as an antibacterial agent. Indole-3-butyric acid is a spare form of auxins in plants and can cause the formation of masses of undifferentiated cells called callus. It may be converted into indole-3-acetic acid through a process similar to the β-oxidation of fatty acids. Their conversion suggests that it works as a storage sink for IAA in plants. There is additional evidence that indicates this compound functions as an auxin on its own but is not converted to IAA [31]. The indole-3-carbinol content ranged between 14.1 μg/g and 212.013 μg/g ADB and was greatest when supplemented with 5 µg/mL of exogenous β-sitosterol, with a 15-fold increase relative to the control. The accumulation of indole-3-butyric acid increased from 16.336 μg/g to 158.009 μg/g ADB, equivalent to a 10-fold increase, when supplemented with 0.25 µg/mL compound (Figure 4, Table S3).

Indole-3-acetic acid increased in the *S. netropsis* IMV Ac-5025 biomass due to the presence of β-sitosterol, but was present in smaller quantities compared to other forms of auxins, from 2.320 μg/g to 18.860 μg/g ADB (Figure 4, Table S3). This is likely due to the fact that extremely high quantities of the active form of auxins can have negative consequences for the bacterial cell. Modified auxin forms—indole-3-acetic acid hydrazide, indole-3-carboxaldehyde, and indole-3-carboxic acid—are used to regulate auxin homeostasis and have been identified in producer biomass. However, very little is known about the integration of multiple auxins’ biosynthesis pathways [31], especially in streptomycetes’ cells. The quantity of auxins increased in the *S. netropsis* IMV Ac-5025 biomass under the action of β-sitosterol.

### 2.4. Biosynthesis of Cytokinins

**Figure 5:**
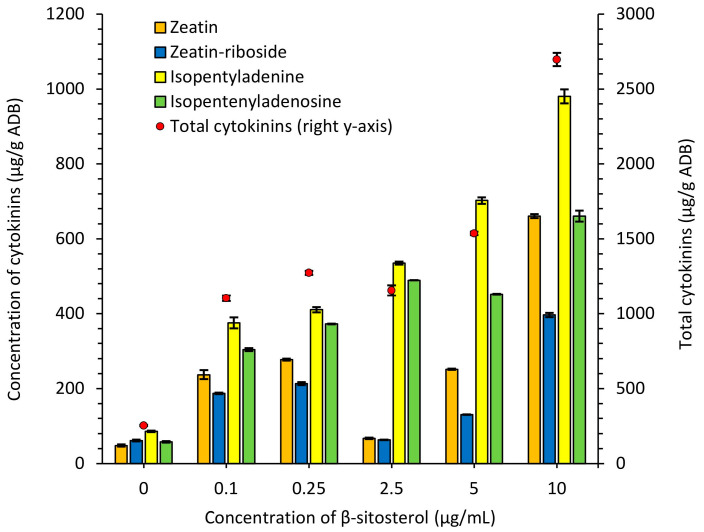
Cytokinins’ biosynthesis in *S. netropsis* IMV Ac-5025 biomass grown in synthetic nutrient medium supplemented with exogenous β-sitosterol. Red circles show the sum of cytokinins. For greater clarity, this parameter is displayed on the secondary axis as these values significantly exceed the number of separated cytokinins.

Cytokinins are known to act as signaling molecules by modulating gene regulatory networks. They are also involved in metabolic processes, differentiation, and secondary metabolite production [32]. Phytohormone active forms, namely zeatin and isopentyladenine, as well as less active zeatin-riboside and isopentenyladenosine, were quantified in the *S. netropsis* IMV Ac-5025 biomass grown in synthetic nutrient medium supplemented with exogenous β-sitosterol. An increase in the accumulation of intracellular cytokinins correlated with an increase in the exogenous compound concentration. For all types of cytokinins, the quantity was highest when 10 µg/mL of β-sitosterol was added. The sum of cytokinins ranged between 253.83 μg/g and 2697.02 μg/g ADB and was highest when supplemented with exogenous sterol, with a 11-fold increase relative to the control (Figure 5, Table S4).

Zeatin is a kind of cytokinin that is known to play important roles in plant growth and development, but its physiological importance in bacteria is limited. There is evidence that it influences the morphological development of streptomycetes, particularly the formation of aerial mycelium and sporulation. It may stimulate or enhance the differentiation of the cells into specialized structures that are crucial for the life cycle [22]. The zeatin content ranged between 48.1 μg/g and 660.34 μg/ ADB, with a 13.7-fold increase relative to the control. Zeatin-riboside is a natural product with less physiological activity compared to zeatin [33]. The accumulation of this metabolite increased from 61.61 μg/g to 396.23 μg/g ADB, equivalent to a 6.4-fold increase, when supplemented with 10 µg/mL of β-sitosterol. Isopentyladenine has diverse effects on bacterial physiology, ranging from morphological development to the regulation of secondary metabolites, cell proliferation and growth, and even stress responses to such factors as nutrient availability, oxidative stress, or osmotic stress via a series of phosphorylation cascades [33]. The isopentyladenine content varied between 86.12 μg/g and 980.13 μg/g ADB, with an 11.4-fold increase relative to the control. Isopentenyladenosine is a hydrocarbyl adenosine in which adenosine is substituted at N-6 by an isopentenyl group. Like other cytokinins, it can potentially affect gene expression and signaling pathways in streptomycetes. The accumulation of this phytohormone in the producer’s biomass increased from 58.01 μg/g to 660.32 μg/g ADB, equivalent to an 11.4-fold increase, when supplemented with 10 µg/mL of β-sitosterol.

## 3. Materials and Methods

### 3.1. Cultivation and Storage of Streptomyces

*S. netropsis* IMV Ac-5025 was isolated from chestnut soils in Ukraine, and has great biosynthetic potential, and is promising for use in biotechnology. The strain is registered in the Microorganisms Depositary of the D.K. Zabolotny Institute of Microbiology and Virology of the National Academy of Sciences of Ukraine, Kyiv, Ukraine [19].

To study the biosynthesis of polyene antibiotics and the accumulation of sterols, phytohormones, and biomass under the action of exogenous β-sitosterol, the strain was grown in submerged conditions. Previously, the strain was stored on oatmeal agar (ISP-3) at 4 °C [34]. Flasks containing nutrient medium (soy broth 15 g/L, yeast extract 5 g/L, glucose 20 g/L, and pH 7.0) were inoculated with *S. netropsis* IMV Ac-5025 and incubated in a shaking incubator for 2 days at 28 °C, 240 revolutions per minute with steady aeration. We added 5% of the received inoculum into glass flasks with the appropriate nutrient media and incubated under the same conditions for the next 7 days. Liquid starch-ammonia medium was used as a synthetic nutrient medium (soluble starch 10 g/L; MgSO_4_ ·7H_2_O 1 g/L; NaCl 1 g/L; (NH_4_)_2_SO_4_ 1 g/L; K_2_HPO_4_ 1 g/L; CaCO_3_ 3 g/L; distilled water 1 L, pH 7.0). Soy nutrient medium was used as an organic nutrient medium (soya broth 12.0 g/L; yeast extract 3.0 g/L; corn extract 3.0 g/L; glucose 70.0 g/L; CaCO_3_—4.5 g/L, K_2_HPO_4_—0.3 g/L, tap water 1 L, pH 7.0). Exogenous β-sitosterol (Sigma-Aldrich, Darmstadt, Germany) was added at the following concentrations: 0.1, 0.25, 2.5, 5, and 10 μg/mL; control cultures were grown without the addition of sterol. The biomass accumulation was calculated at the stationary phase of growth using gravimetric methods and expressed in grams of absolutely dry biomass (ADB) or in grams per liter of culture medium [35]. Polyene antibiotics, sterols, and phytohormones were quantified at this stage.

### 3.2. Determination of Polyene Antibiotics by Spectrodensitometric Thin-Layer Chromatography (TLC)

The polyene antibiotics were extracted from the wet producer biomass by 96% (*v*/*v*) aqueous ethanol for 24 h. Ethanol extracts were applied on chromatographic plates of TLC Silica gel 60 F254 (Merck, Darmstadt, Germany). The chromatography of the extracts was carried out in a system of solvents: butanol:acetic acid:water (3:1:1). The spots formed on the chromatographic plates were compared with the control. Prepurified heptaene candidine and tetraene compounds synthesized by *S. netropsis* IMV Ac-5025 were used as controls. Purification of the compounds was performed through chromatographic separation on chromatographic plates, TLC Silica Gel 60 F254 (Merck, Darmstadt, Germany) in a solution system for polyene antibiotics. The spots formed at the appropriate height of the plate were analyzed using a Spectrodensitometer (Sorbfill TLC View, Kyiv, Ukraine) with the UV filter, with a wavelength of 365 nm, using standard software and identified as corresponding to each of the fractions. The software responded to the height of the peak, which corresponded to candidine or tetraene compounds. The peak area indicated the amount of antibiotic and was supplemented with a control [36,37].

### 3.3. Determination of Sterols Using Gas Chromatography (GC)

Gas chromatography was employed in the determination of the content of sterols in dry biomass of *S. netropsis* IMV Ac-5025. Samples were prepared by adding methanol and a 60% (*w*/*v*) KOH solution before being heated to 80 °C for 2 h using a water bath. Once the reaction mixture had cooled to room temperature, distilled water and dichloromethane were added to separate the hydrophilic and hydrophobic phases. The lower layer of lipids was taken for quantitative and qualitative analysis [38]. Derivatization was performed by adding to the dried samples 100 μL of pyridine (for dissolution) and 100 μL of the reaction mixture: N, O-bis(trimethylsilyl)trifluoroacetamide:trimethylchlorosilane (5:1 (*v*/*v*)). The samples were kept at 70 °C for 30 min. As part of the analysis, selected sterol fractions were determined on a HP 6890 gas chromatograph (Hewlett-Packard, Palo Alto, CA, USA) equipped with a split injector (split splitter), a column thermostat with temperature setting, a flame ionization detector, and special software for peak identification (Chemstation Ver.A.06.03), using an automatic split injector. Column thermostat program: 250 °C (0 min), 15 °C/min → 280 °C (1 min), 15 °C (1 min) → 340 °C (4 min), 0 °C (1 min) → 350 °C (1 min). The injection system with flow splitting contained the following characteristics: temperature—340 °C; pressure—20.75 psi; split ratio—20:1; flow of division—49.9 mL/min; total flow—55.3 mL/min. The samples are carried by a moving carrier gas stream of helium; carrier gas flow—2.5 mL/min. The capillary column was Vf-5 ms with a stationary non-polar phase of 5% phenyl-dimethylpolysiloxane: length—0 mm, diameter—0.32 mm, film thickness—0.25 μm, high temperature (maximum temperature was 350 °C). Flame ionization detector—350 °C; hydrogen flow—30 mL/min; air flow—300 mL/min; auxiliary flow—20 mL/min; the auxiliary carrier gas was nitrogen. The volume of the injected sample was 1.0 μL, the concentration of the samples was 1.0 mg/mL, and the total analysis time was 11.0 min. Sterol fractions were identified by retention time according to the retention time of the standard (reference) sterol mixture in the composition: squalene, ergosterol, cholesterol, stigmasterol, β-sitosterol, and 24-epibrassinolide (Sigma-Aldrich, Germany).

### 3.4. Determination of Auxins and Cytokinins Using High-Performance Liquid Chromatography (HPLC)

Preliminary purification and concentration of phytohormones extracted from *S. netropsis* IMV Ac-5025 biomass were carried out through thin-layer chromatography. For this purpose, the purification of compounds was performed in three stages in a mixture of solvents used sequentially: chloroform (the first step), 12.5% ammonia (the second step), and ethyl acetate: acetic acid (20:1) (the third step). The second step of purification involved the quantitative application of both pure cytokinin (zeatin) and auxin (indole-3-acetic acid) and samples on chromatographic plates for the separation of the following compounds. Then, for each sample, a silica gel layer corresponding to cytokinins and indole compounds was removed from the plate and transferred into tubes. Cytokinins’ extraction was carried out using 96% aqueous ethanol and auxins with ethyl acetate. Samples were kept at 4 °C for 1 day. The samples were then shaken and centrifuged. The obtained supernatant was transferred into smaller tubes and left for 1 day in a thermostat at 28 °C for the phytohormones’ evaporation and concentration. The compound was quantitatively washed from the tube walls using the appropriate solvents on the third day (as described above) [39]. Samples were analyzed by high-performance liquid chromatography (HPLC), using an Agilent 1200 liquid chromatograph (Agilent Technologies, Santa Clara, CA, USA) and an Agilent G1956B mass spectrometry (MS) detector. The separation was carried out by the analytical column Zorbax SB-C18 (2.1 mm × 150 mm, 3 μm; Agilent Technologies, USA), and the flow rate of the mobile phase was 0.35 mL/min. The volumes of injections were 3 μL. Columns were kept at 30 °C. Elution was carried out in gradient mode with acetonitrile (A)—0.1% water + formic acid (B): 20% A for 5 min, then the concentration of A was increased from 20% to 80% over 10 min, followed by an increase to 100% over 0.5 min. Elution continued for a further 8.5 min. Mass spectrometric analysis recorded positive and negative ions in the ratio m/z. The calculation of chromatograms was performed using the Agilent Chemstation Ver.A.06.03 software. HPLC/MS analysis of the ethanolic extracts of *S. netropsis* IMV Ac-5025 was performed at the Laboratory of Biological Polymer Compounds, D.K. Zabolotny Institute of Microbiology and Virology, NAS of Ukraine. Phytohormones analysis was performed using chemically pure auxins: indole-3-acetic acid (IAA), indole-3-acetic acid hydrazide, indole-3-carbinol, indole-3-butyric acid, indole-3-carboxaldehyde, indole-3-carboxylic acid (Sigma-Aldrich, Germany) and cytokinins: zeatin, zeatin riboside, N 6-(Δ 2-Isopentenyl) adenine, N6-isopentenyladenosine (Sigma-Aldrich, Germany).

All analyses were carried out in three replicates, and then the standard deviation (± SD) and average values (x) were calculated.

## 4. Discussion

Analyzing the metabolism of biologically active compounds derived from bacteria provides insights into the signaling and biosynthetic pathways that underlie these processes. This is especially important for industrially valuable strains that produce natural products with practical applications. *S. netropsis* IMV Ac-5025 was isolated from the soil and studied in order to characterize its biosynthetic ability and explore interactions with the regulatory network for the biosynthesis of polyene antibiotics and other secondary metabolites. We demonstrated that biosynthetic pathways are activated, resulting in increased biomass accumulation and increased production of polyene antibiotics, sterols, and phytohormones when β-sitosterol is added to the culture medium.

Sterols play an important role in modeling stress responses through their influence on the biomembranes of bacteria, plants, and animals [40]. In a variety of yeast species, sterols can regulate the cellular processes associated with hypoxia, osmotic stress, and other conditions [41]. We also do not exclude the possibility that the effects observed by the supplementation of β-sitosterol can be partially explained as a non-specific reaction to stress. However, the activation of biosynthetic pathways with the addition of exogenic β-sitosterol to the nutrient medium reveals its influence on a complex of physiological reactions. This influence on membrane function and stability could be essential for sustaining bacterial stress responses [42]. In some bacteria, hopanoides, a type of sterol, provide adaptation to extreme environmental conditions. They are synthesized in the aerial hyphae and spores of streptomycetes and help to minimize the loss of water through the membrane into the air [9,10].

The data in this study support the hypothesis that there are indirect interactions between the biosynthetic pathways of polyene antibiotics and sterols. Polyene antibiotics are synthesized in several stages, macrolide rings form, an aromatic fragment, and the aggregation of an amino sugar fragment [43]. Sterols are formed from more than 30 different reactions, which can be grouped into several stages: the biosynthesis of mevalonic acid, isopentenyl diphosphate, farnesyl diphosphate, and squalene formation, an intermediate product in the biosynthesis of sterols, hopanoids, and related pentacyclic triterpenes in bacteria. Mevalonate groups are also involved in polyketide molecule biosynthesis, which suggests that polyene antibiotics and sterols are indirectly related to each other. Other studies have also shown that mevalonic acid, a precursor of sterol molecules, stimulates the action of the tetraene antibiotic antimycon [7,43]. This may support the hypothesis that polyene antibiotics and sterol biosynthesis pathways are indirectly linked.

While the biosynthesis and function of sterols in bacteria have received relatively little exploration, reports indicate that certain sterols undergo additional chemical modifications during this process. For example, methanotrophic bacteria modify sterols into distinct monomethylated structures specific to *Methylococcaceae* [7], sterol-producing *Planctomycetes* conjugate sterols to an unidentified macromolecule, and several *Myxococcota* produce intermediates in the cholesterol biosynthesis pathway, including zymosterol. Furthermore, the biological functions of sterols are also important in a variety of physiological processes, including cell signaling, membrane homeostasis, and more. For example, these chemical modifications in eukaryotes serve as a branching point for downstream metabolite biosynthesis. Oxidation reactions are involved in converting sterols into a wide range of compounds, such as oxysterols, bile acids, steroid hormones, and brassinosteroids, all functioning as ligands in signaling pathways. Eukaryotes also conjugate sterols to sugars, proteins, and other lipids, further expanding their functions to include cell defense, energy storage, digestion, and signaling [8]. We hypothesize that both native, cell-modified, and transported β-sitosterol played a significant signaling role in affecting the network of biosynthetic pathways in *S. netropsis* IMV Ac-5025. The question arises whether exogenous sterols penetrate the mycelium of streptomycetes. With some probability, we can support the idea that they do, given that sterol transporter proteins are found in *Methylococcus capsulatus.* It produces specific proteins with transport properties in their inner membranes, periplasm, and outer membranes, serving as conduits for transporting modified sterols [42]. In addition, we noted that enriching the nutrient medium with exogenous β-sitosterol led to the accumulation of squalene and various sterols, including ergosterol, cholesterol, stigmasterol, β-sitosterol, and 24-epibrassinolide, which serve as intermediates in the biosynthesis of end products. It is known that sterol intermediates play a regulatory role in lipid homeostasis [44], biosynthesis of subsequent products [1], and stress response [45]. Various intermediates in sterol compound biosynthesis, such as desmosterol and zymosterol, are hypothesized to act as functional lipids in the formation of the final product, cholesterol [8]. Based on the data from the literature and the experimental results presented in this study, we can infer that the increased accumulation of sterol compounds also assumes a regulatory role in the biosynthesis of various natural products, particularly antibiotics. Polyene antibiotics are intracellular secondary metabolites, but they were released from the cell in response to exogenous β-sitosterol. This phenomenon is most likely a protective mechanism that prevents streptomycetes’ cells from reaching their high concentrations. Furthermore, it is possible to observe an interaction with membrane sterols inside the producer cells, resulting in an increase in the permeability of the cell wall [30,46]. We assume that exogenous β-sitosterol and its effects on *S. netropsis* IMV-Ac 5025 may be involved in the processes listed above, leading to the activation of numerous biosynthetic processes, including biomass accumulation and the stimulation of antibiotic, sterol, and phytohormone biosynthesis. Increasing the yield of these natural products in turn causes complex changes in cell metabolism.

Auxins are involved in regulating the metabolism of higher plants at small concentrations. However, it is largely unknown how auxins perform their regulatory functions in microorganisms, despite the fact that their importance as global signal molecules is being increasingly recognized. Remarkably, a growing body of research supports the notion that auxins are crucial signal molecules in bacteria, regulating processes including stress resistance, antibiotic biosynthesis, virulence factors, and nutrient transport [34]. Different microorganisms metabolize auxins and use them for energy and nutrition. Similarly, in streptomycetes, auxins might influence gene expression and regulate the metabolic pathways involved in primary and secondary metabolism. This may involve interactions with regulatory networks related to nutrient utilization and carbon metabolism. Also, the biosynthesis of polyene antibiotics and auxins are indirectly related through the shikimate pathway in streptomycetes. It is a specialized pathway for the biosynthesis of benzoic aromatic compounds, which gathers the main building blocks needed to build aromatic amino acids (phenylalanine, tyrosine, and tryptophan) in bacteria. It is functionally related to acetate malonate, which is involved in the construction of the benzene nuclei of polyketides required for the closure (zip-assembly) of aromatic nuclei and for the construction of auxin molecules [37,47,48].

Crosstalk between cytokinin and sterol pathway biosynthesis in streptomycetes requires further study to determine the molecular mechanisms involved. It is believed that the endogenous cytokinins produced by streptomycetes may regulate their own growth, development, and secondary metabolism through various mechanisms, which are still under investigation. Some antibiotics are also enhanced by cytokinin signaling. It is worth noting that the the physiological functions of cytokinins in representatives of the *Streptomyces* genus is an emerging field of study, and more detailed research results may shed light on their role in the future [49]. Some studies have demonstrated a crosstalk between brassinosteroids (polyhydroxylated steroidal phytohormones) and cytokinins with respect to plant growth. They interact to regulate plant growth and development, synergistically promoting cell division and differentiation. Furthermore, they may regulate the expression of genes involved in plant growth and development. Therefore, the efflux of sterols can affect membrane properties and modulate cytokinin receptor function and signaling [50,51,52]. Thus, we can potentially assume the presence of an analagous mechanism in *S. netropsis* IMV Ac-5025 by affecting the membrane composition or signaling pathways. The addition of β-sitosterol leads to both the accumulation of cytokinins and cell division, as evidenced by the increase in biomass.

In summary, these results provide new insights into the interactions of polyene antibiotics, sterols, and phytohormones in the biosynthetic pathways of *Streptomyces* bacteria, laying the foundation for future research on their metabolome. The theoretically established pattern between the biosynthesis of antibiotics and steroid compounds by *S. netropsis* IMV Ac-5025 was confirmed experimentally. Exogenous β-sitosterol stimulated the accumulation of polyene compounds, such as heptaene candidine and tetraene polyene antibiotics, and caused their excretion from the cells into the culture supernatant. At the same time, there was an increase in the intracellular accumulation of sterols, as well as phytohormones, which have physiological activity under the action of an exogenous compound.

## 5. Conclusions

The obtained results confirm the hypothesis formulated earlier by the authors that β-sitosterol has a regulatory role and affects the streptomycetes’ secondary metabolite pathways; namely, polyene antibiotics, sterols, auxins, and cytokinins. Also, this compound has a positive effect on cell division, which is confirmed by the growth of biomass. In soil streptomycetes, these regularities in physiology and biochemistry are novel and prospective, but the precise mechanisms remain unknown and require further study. To expand our knowledge of the metabolome of streptomycetes, it will be necessary to study the mechanisms underpinning the signalling role that sterols play in the biosynthesis of polyene antibiotics or other types of secondary metabolites. The results of this study can be applied to microbial biotechnology for the production of bioactive metabolites with useful properties from biomass extracts and culture liquids. For example, polyene antibiotics can be used as bioproducts for plant treatment to protect against fungal phytopathogens or as biostimulants used to improve growth and development by secretion of phytohormones. Such bioproducts are widely used both in Ukraine and around the world; they are non-toxic, ecological, and safe, and they are used as an alternative to chemical pesticides. Overall, this study sheds light on new physiological aspects of the strain and new properties of sterol on secondary metabolite production and, concomitantly, emphasizes the industrial interest of *S. netropsis* IMV Ac-5025 by showing its potential phytoprotective and plant growth-stimulating effects.

## Data Availability

The data presented in this study are available on request from the corresponding author.

## Supplementary Materials

The following supporting information can be downloaded at: https://www.mdpi.com/article/10.3390/antibiotics13020146/s1, Table S1. Polyene antibiotics’ accumulation by *S. netropsis* IMV Ac-5025 grown in synthetic and organic nutrient media in culture liquid supplemented with exogenous β-sitosterol (ADB—absolutely dry biomass); Table S2. Biosynthesis of squalene and sterols in *S. netropsis* IMV Ac-5025 biomass grown in synthetic nutrient medium supplemented with exogenous β-sitosterol; Table S3. Biosynthesis of auxins in *S. netropsis* IMV Ac-5025 biomass grown in synthetic nutrient medium supplemented with exogenous β-sitosterol, Table S4. Cytokinins’ biosynthesis in *S. netropsis* IMV Ac-5025 biomass grown in synthetic nutrient medium supplemented with exogenous β-sitosterol.
