# Supplementary material for: Natural Products Biosynthesis by Streptomyces netropsis IMV Ac-5025 under Exogenous Sterol Action
Source: Antibiotics (Basel). 2024 Feb 1;13(2):146. doi: 10.3390/antibiotics13020146 (PMC10886242; doi:10.3390/antibiotics13020146)
Supplement: Supplementary file 1 [file antibiotics-13-00146-s001.zip › antibiotics-2843329-supplementary.pdf]

Supplementary materials

Natural products biosynthesis by *Streptomyces netropsis* IMV Ac-5025 under exogenous sterol action

Mariia Loboda [1], Liudmyla Biliavska [1], Halyna Iutynska [1], Jake Newitt [2] and Ruslan Mariychuk [3,*]

[1] Department of General and Soil Microbiology, D.K. Zabolotny Institute of Microbiology and Virology, National Academy of Sciences of Ukraine, Akademika Zabolotnoho str., 154, Kyiv, Ukraine; marichka20loboda@gmail.com (M.L.), bilyuvskal@ukr.net (L.B.), galyna.iutynska@gmail.com (H.I.)

[2] Department of Molecular Microbiology, John Innes Centre, Norwich NR4 7UH, United Kingdom; jake.newitt@jic.ac.uk

[3] University of Presov, Presov, Slovakia; ruslan.mariychuk@unipo.sk

* Correspondence: ruslan.mariychuk@unipo.sk

Table S1. Polyene antibiotics' accumulation by *S. netropsis* IMV Ac-5025 grown in synthetic and organic nutrient media in culture liquid supplemented with exogenous β-sitosterol (ADB – absolutely dry biomass).

Table S2. Biosynthesis of squalene and sterols in *S. netropsis* IMV Ac-5025 biomass grown in synthetic nutrient medium supplemented with exogenous β-sitosterol.

Table S3. Biosynthesis of auxins in *S. netropsis* IMV Ac-5025 biomass grown in synthetic nutrient medium supplemented with exogenous β-sitosterol.

Table S4. Cytokinins' biosynthesis in *S. netropsis* IMV Ac-5025 biomass grown in synthetic nutrient medium supplemented with exogenous β-sitosterol.

Table S1. Polyene antibiotics′ accumulation by *S. netropsis* IMV Ac-5025 grown in synthetic and organic nutrient media in culture liquid supplemented with exogenous β-sitosterol (ADB – absolutely dry biomass).

Syntetic nutrient medium							
β-Sitosterol, μg/mL	Culture liquid			Biomass, g/L	Biomass extract		
	Tetraene, μg/mL	Candidine, μg/mL	Sum of antibiotics, μg/mL		Tetraene, mg/g ADB	Candidine, mg/g ADB	Sum of antibiotics, mg/g ADB
0	2.68±0.09	0.32±0.07	3±0.16	2.65±0.2	1.399±0.11	0.53±0.01	1.929±0.1
0.1	7.3±0.41	0.5±0.06	7.8±0.46	3.7±0.23	1.543±0.04	0.772±0.01	2.315±0.04
0.25	7.12±0.1	3.1±0.41	10.22±0.36	3.29±0.23	1.556±0.13	0.809±0.03	2.365±0.11
2.5	7.5±0.06	3.05±0.16	10.55±0.12	4.44±0.24	2.405±0.23	0.907±0.11	3.312±0.3
5	7.66±0.25	3.9±0.57	11.56±0.32	5.2±0.2	2.442±0.26	1.326±0.02	3.768±0.29
10	8.4±0.41	4.98±0.07	13.38±0.47	4.56±0.2	3.218±0.12	1.699±0.1	4.919±0.22
**Organic nutrient medium**							
β-Sitosterol, μg/mL	Culture liquid			Biomass, g/L	Biomass extract		
	Tetraene, μg/mL	Candidine, μg/mL	Sum of antibiotics, μg/mL		Tetraene, mg/g ADB	Candidine, mg/g ADB	Sum of antibiotics, mg/g ADB
0	67±1.2	41±1.49	108±2.54	7.15±0.25	6.5±0.29	2.71±0.11	9.21±0.4
0.1	73±1.13	44±1.15	117±0.8	7.34±0.34	8.3±0.53	3.8±0.15	12.1±0.66
0.25	83±0.57	43±1.49	126±0.96	8.58±0.4	9.3±0.38	4±0.26	13.3±0.63
2.5	87±1.7	30±1.41	120±2.89	9.54±0.32	4.8±0.16	2.87±0.12	7.67±0.27
5	90±1.76	31±1.28	121±2	10.16±0.25	10±0.41	7.7±0.11	17.7±0.45
10	106±1.18	39±1.08	145±1.93	10.1±0.3	12.4±0.5	8.4±0.33	20.8±0.26

Table S2. Biosynthesis of squalene and sterols in *S. netropsis* IMV Ac-5025 biomass grown in synthetic nutrient medium supplemented with exogenous β-sitosterol.

β-Sitosterol, µg/mL	Compounds, mg/g of absolutely dry biomass						
	Squalene	Ergosterol	Cholesterol	Stigmasterol	β-sitosterol	24-Epi-brassinolide	Total squalen and sterols
0	0.10 ±0.01	0.05 ±0.01	0.03± 0.01	0.91 ±0.32	0.51 ±0.24	0.15 ±0.01	1.65 ±0.43
0.1	0.32 ±.0.18	0.08 ±0.01	0.05 ±0.01	1.82 ±0.45	0.71 ±0.28	0.21 ±0.01	3.18 ±0.59
0.25	0.22 ±0.15	0.05 ±0.01	0.11 ±0.01	2.73 ±0.55	1.12 ±0.235	0.52 ±0.24	4.75 ±0.72
2.5	0.13 ±0.12	0.08 ±0.01	0.08 ±0.01	2.25 ±0.5	0.83 ±0.30	0.56 ±0.25	3.92 ±0.66
5	0.24 ±0.16	0.12 ±0.01	0.13 ±0.02	2.42 ±0.52	0.63 ±0.26	0.36 ±0.20	3.90 ±0.66
10	0.55 ±0.24	0.36 ±0.20	0.23 ±0.16	0.74 ±0.29	0.96 ±0.32	0.26 ±0.01	3.10 ±0.59

Table S3. Biosynthesis of auxins in *S. netropsis* IMV Ac-5025 biomass grown in synthetic nutrient medium supplemented with exogenous β-sitosterol.

β-Sitosterol, µg/mL	Auxins, µg/g of absolutely dry biomass						
	Indole-3-acetic acid	Indole-3-acetic acid hydrazide	Indole-3-butiric acid	Indole-3-carbinol	Indole-3-carboxaldehyde	Indole-3-carboxylic acid	Sum of auxins
0	2.320±0.51	3.168±0.59	16.336±1.34	14.100±1.25	4.320±0.69	34.000±1.94	74.244±2.83
0.1	18.860±1.45	2.208±0.50	86.480±3.10	26.220±1.71	12.000±1.15	61.140±2.61	206.908±4.79
0.25	15.700±1.32	2.001±0.47	158.009±4.19	64.000±2.67	14.000±1.25	38.000±2.05	291.710±5.69
2.5	8.750±0.99	4.500±0.7	42.000±2.16	165.000±4.28	24.000±1.63	77.500±2.93	321.750±5.98
5	8.120±0.95	13.668±1.23	92.280±3.20	212.013±4.85	28.600±1.78	100.120±3.34	454.801±7.11
10	5.841±1.95	3.000±0.58	99.120±3.32	70.001±2.79	64.310±2.67	117.999±3.62	360.271±6.33

Table S4. Cytokinins' biosynthesis in *S. netropsis* IMV Ac-5025 biomass grown in synthetic nutrient medium supplemented with exogenous β-sitosterol.

β-Sitosterol, μg/mL	Cytokinins, μg/g of absolutely dry biomass				
	Zeatin	Zeatin-riboside	Isopentyladenine	Isopentenyladenosine	Cytokinins' sum
0	48.093±0.58	61.612±2.45	86.121±2.26	58.007±2.21	253.833±4.51
0.1	237.016±2.38	187.044±2.4	375.211±16.58	303.783±4.28	1103.054±19.94
0.25	277.126±0.54	213.211±4.37	410.222±7.85	372.393±0.99	1272.952±14.22
2.5	67.215±0.34	63.211±0.96	535.041±4.65	489.522±0.66	1154.989±4.06
5	251.412±0.32	130.523±0.85	702.018±9.77	451.899±1.66	1535.852±13.47
10	660.336±1.08	396.225±6.39	980.135±21.26	660.319±16.72	2697.015±38.6
